# Serotonin transporter deficiency, but not absence of platelet serotonin, impairs thrombus formation in a model of deep vein thrombosis

**DOI:** 10.1016/j.rpth.2025.102970

**Published:** 2025-07-15

**Authors:** Katharina Naber, Maximilian Mauler, Nancy Schanze, Pia Kröning, Daniela Stallmann, Daniel Duerschmied, Dirk Westermann, Nadine Gauchel

**Affiliations:** 1 Department of Cardiology and Angiology, University Heart Center Freiburg-Bad Krozingen, Medical Center - University of Freiburg, Freiburg, Germany; 2 Department of Obstetrics and Gynecology, University Medical Center Freiburg, University of Freiburg, Freiburg, Germany; 3 Faculty of Medicine, University of Freiburg, Freiburg, Germany; 4 Department of Cardiology, Haemostaseology, and Medical Intensive Care, Medical Centre Mannheim, Medical Faculty Mannheim, Heidelberg University, Mannheim, Germany; 5 European Center for AngioScience, German Centre for Cardiovascular Research partner site Heidelberg/Mannheim, Centre for Cardiovascular Acute Medicine Mannheim, Medical Centre Mannheim, Medical Faculty Mannheim, Heidelberg University, Mannheim, Germany; 6 Helmholtz-Institute for Translational AngioCardioScience, Max Delbrück Center for Molecular Medicine, Helmholtz Association, Heidelberg University, Heidelberg, Germany

**Keywords:** deep vein thrombosis, platelets, serotonin, serotonin transporter, thromboinflammation

## Abstract

**Background:**

The majority of peripheral serotonin is stored in dense granules of circulating blood platelets and released upon platelet activation. Recently, an immunomodulatory role of serotonin in inflammation has been found, influencing the recruitment of leukocytes, especially neutrophils.

**Objectives:**

Since deep vein thrombosis creates an inflammatory milieu, called thromboinflammation, this study examined the impact of peripheral platelet serotonin on the development of venous thrombosis.

**Methods:**

To induce deep vein thrombosis, a stenosis model of the inferior vena cava was used. Six- to 8-week-old C57BL/6 (wild-type [WT]), selective serotonin reuptake inhibitor-treated C57BL/6 (depleted serotonin pools in platelets), serotonin transporter knockout (SERT^-/-^), and tryptophan hydroxylase 1 knockout (Tph1^-/-^) mice were used. Thrombus volume was measured, and its composition was analyzed after 48 hours using immunofluorescence microscopy. Neutrophils and platelet-neutrophil complexes were analyzed using flow cytometry.

**Results:**

SERT^-/-^ mice formed significantly fewer and smaller thrombi compared with WT (mean ± SD, 1.09 mm^3^ ± 2.53 vs 13.1 mm^3^ ± 11.1; *P* = .002) and Tph1^-/-^ mice (1.09 mm^3^ ± 2.53 vs 11.3 mm^3^ ± 6.84; *P* = .02) and had lower levels of neutrophils in the blood. Thrombi in the WT and Tph1^-/-^ groups were comparable. In SERT^-/-^ mice, there was no decrease in circulating platelet-neutrophil complexes.

**Conclusion:**

The extent of venous thrombosis did not depend on peripheral serotonin in our mouse model, but rather on the presence of the serotonin transporter. In the absence of the serotonin transporter, the thrombogenic property as well as the overall immune response to venous thrombosis was reduced. The distribution of the serotonin transporter on immune cells and its thrombogenic potential should be studied further.

## Essentials

• Platelet serotonin may affect thrombus formation by influencing immune response and inflammation.
• Vena cava stenosis in mice was used to study how serotonin affects thrombus formation.
• Mice lacking the serotonin transporter had fewer thrombi and less immune cell activity.
• Thrombus size was linked to the serotonin transporter, not to serotonin levels, in this study.

## 1 Introduction

Venous thromboembolism is one of the most common cardiovascular diseases. The clinical manifestation is mostly deep vein thrombosis, including potentially serious complications such as pulmonary artery embolism [1]. In addition to this serious disease, an immune response to pathogens sometimes leads to thrombogenesis in the venous system, resulting in immunothrombosis [2].

Specifically, immunothrombosis is an interaction between platelets and leukocytes. They interact by cross-activating, expressing, and binding to various surface receptors in addition to extracellular components such as fibrinogen or neutrophil extracellular traps (NETs) [[2], [3], [4], [5]]. This creates an inflammatory environment by releasing various mediators, which is referred to as thromboinflammation.

One of the many mediators released from platelet granules upon activation is serotonin [6,7]. Although its exact role in venous thrombogenesis remains unclear, it is known that serotonin itself can activate platelets, induce neutrophil degranulation, and increase CD11b expression on neutrophils [[7], [8], [9]].

Serotonin is found both in the central nervous system as a neurotransmitter and in the peripheral blood, where it is released into the circulation after being synthesized by the rate-limiting enzyme tryptophan hydroxylase (Tph) 1 in enterochromaffin cells [10]. The central serotonin is synthesized by a different isoform of the enzyme, Tph2 [11].

More than 90% of peripheral serotonin is taken up by serotonin transporter (SERT) into platelets and stored in platelet dense granules [7]. Platelets themselves are not able to synthesize serotonin. When platelets get activated upon external stimulation, internal contents of their granules, including high amounts of serotonin, are released [12]. Interactions with surrounding cells occur, such as activation of additional platelets and endothelium, vasoconstriction, and recruitment of leukocytes, within the context of inflammation [8,13]. It is known that monocytes, macrophages, mast cells, T cells, and endothelium express Tph1 and are able to synthesize serotonin [14,15]. Serotonin receptors are found on many immunomodulatory cells, including leukocytes, platelets, and mast cells, as well as on endothelial cells and vascular smooth muscle cells [14].

The aim of this study was to evaluate the impact of platelet serotonin on the development of deep vein thrombosis in a mouse model. Therefore, we analyzed the effect of peripheral serotonin on leukocyte recruitment, thrombus growth, and thrombus composition in a stenosis model of deep vein thrombosis in mice. To generate different serotonin levels, we used wild-type (WT) mice, Tph1 knockout (Tph1^-/-^) mice, SERT knockout (SERT^-/-^) mice, and WT mice fed with the selective serotonin reuptake inhibitor (SERT inhibitor) fluoxetine (Flx).

Tph1^-/-^ mice are not able to synthesize serotonin in the periphery at all [11]. Therefore, platelets cannot store or release serotonin, which leads to a depletion of serotonin in plasma and serum. In SERT^-/-^ mice, platelets cannot take up serotonin and store it in their dense granules, which leads to a lack of release of serotonin upon activation of platelets. In contrast to Tph1^-/-^ mice, cells expressing Tph1 in SERT^-/-^ mice can synthesize and release serotonin (such as mast cells, endothelium, monocytes, and macrophages). This may lead to lower, but measurable, serotonin levels in plasma. The SERT inhibitor Flx mimics the pharmacologic effects of SERT^-/-^ mice and depletes platelet serotonin [13].

## 2 Methods

### 2.1 Animals

Six- to 8-week-old male C57BL/6 mice (WT) were obtained from Charles River Laboratories and housed in the local animal facility. Tph1^-/-^ [11] and SERT^-/-^ mice were on C57BL/6J background and were bred in the local animal facility. Tph1^-/-^ mice were kindly provided by Michael Bader from the Max Delbrück Center for Molecular Medicine (Berlin, Germany). All animal experiments were performed in compliance with the German Animal Protection Law. The mice were housed and handled in accordance with good animal practice as defined by Federation of European Laboratory Animal Science Associations (FELASA; www.felasa.eu) and the national animal welfare body Gesellschaft für Versuchstierkunde / Society of Laboratory Animal Science (GV-SOLAS; www.gv-solas.de). The Animal Welfare Committee of the University of Freiburg, as well as the local authorities (Regierungspräsidium Freiburg), approved all animal experiments (file #35-9185.81/G-19/139).

### 2.2 Pharmacologic SERT blocking

WT mice received drinking water supplemented with the selective serotonin reuptake inhibitor Flx (daily dose of 10 mg/kg, LKT Laboratories) over a time period of 21 days to deplete platelet serotonin pools, as described before [13].

### 2.3 Mouse model of stenosis-induced inferior vena cava thrombosis

The stenosis-induced model of venous thrombosis was performed as previously described [16]. In brief, mice were anesthetized with 4% isoflurane for induction and 2.5% isoflurane via a nose cone during surgery. Mice were placed on a 37 °C heating pad. A midline laparotomy was performed. The small bowel was exteriorized and placed on a moistened gauze pad to the animal’s left. The infrarenal inferior vena cava (IVC) was exposed and all visible side and back branches ligated with a 7-0 Prolene suture. To induce stenosis and not stasis, a 7-0 Prolene suture was tied down over a 30-gauge needle on the IVC, caudal to the left renal vein, and the needle was then removed cautiously, without piercing the vessel or any other organ. The small bowel was placed back, and the peritoneum and skin were closed.

Control animals did not undergo sham surgery to reduce stress on these animals, in line with good animal practice, as we primarily wanted to focus on the different thrombus formation between the different mouse groups with varying platelet serotonin concentrations.

### 2.4 Tissue harvesting

Mice were euthanized 48 hours after thrombus induction, and a cardiac puncture, using a 30-gauge needle coated with unfractionated heparin (B. Braun Melsungen AG), was performed to obtain whole blood. Whole blood was anticoagulated using enoxaparin (1.5 µg/µL, Sanofi). The IVC, containing thrombus, was exteriorized for analysis. Thrombus volume was determined from photographs taken immediately after externalization of the IVC using ImageJ (National Institutes of Health). Thrombus weight, including the surrounding IVC, was measured.

### 2.5 Serotonin concentration in plasma

Anticoagulated blood was incubated with prostacyclin (1 μL in 1 mL, 5 minutes, Cayman) to inhibit platelet activation. After centrifugation (5 minutes, 600 × g), the supernatant was again centrifuged (5 minutes, 1000 × g) and subsequently collected and stored at −20 °C. Plasma serotonin was measured using the Serotonin ELISA Fast Track (Labor Diagnostika Nord) following manufacturer’s instructions. Acquired data were analyzed with SoftMax Pro 7.0 software (Molecular Devices).

### 2.6 Flow cytometry

A total of 100 μL of anticoagulated blood was diluted with 500 μL phosphate buffered saline (PBS) containing 0.9 mmol/L calcium, 0.5 mmol/L magnesium, and 0.1% bovine serum albumin (PBS+/+, bovine serum albumin). A volume of 5 μL of this dilution was incubated with 95 μL lysis buffer (BD Biosciences) for 5 minutes, and leukocytes were counted in a Neubauer chamber. Diluted blood (90 μL) was incubated with 10 μL of antibody mix (see below; 15 minutes in the dark at room temperature). Stained whole blood samples were gently mixed with 400 μL of warm (37 °C) 1× Phosflow Lyse/Fix Buffer (BD Biosciences) and incubated for 20 minutes at room temperature. Data were acquired on a BD FACSCanto II (BD Biosciences) and analyzed with FlowJo v10 software (gating strategies see Supplementary).

The following antibodies were used to evaluate leukocyte subsets and platelet-neutrophil complexes: anti-CD45.2-Amcyan (clone 104), anti-CD206-Pacific Blue (clone C068C2), anti-CD11b-APC/Cy7 (clone M1/70), anti-CD3-FITC (clone 145-2C11), anti-CD19-PE/Cy7 (clone 6D5), anti-Ly6C-PerCP/Cy5.5 (clone HK1.4), anti-lymphocyte antigen 6 complex locus G6D (Ly6G)-PE/Cy7 (clone 1A8), anti-CD11a/CD18-PE (clone H155-78), anti-CD206-PerCP/Cy5.5 (clone C068C2), and anti-CD11b-APC (clone M1/70), all from BioLegend; anti-CD115-APC (clone c-fms) and anti-CD41a-FITC (clone MWReg30), both from Thermo Fisher Scientific; anti-F4/80-PE (clone T45-2342; BD Biosciences), and anti-CD162-PacBlue (clone 2PH1; Beckton Dickinson).

### 2.7 Tail bleeding assay

Tail bleeding time in all groups of mice was assessed without prior thrombus induction. The tail bleeding assay was performed as previously described [17]. In brief, mice were anesthetized with isoflurane (2.5%). The tail was kept in warm saline (37 °C) for 5 minutes to induce vasodilatation. Afterward, 10 mm of the tail tip was amputated with a scalpel, and the tail was submerged in the prewarmed saline again. The time to bleeding cessation was recorded up to 20 minutes. Mice were euthanized at the end of the experiment by neck dislocation.

### 2.8 Microscopy

After tissue collection, the thrombus was preserved in optimal cutting temperature compound (Sakura Finetek Europe B.V.) and stored at −20 °C. Ten-micron sections were cut using a cryostat (Leica) and placed on Superfrost slides (R. Langenbrinck GmbH). These were air-dried and stored at −20 °C. Samples were fixed with 100% acetone for 10 minutes at −20 °C. The samples were then rinsed twice for 5 minutes with PBS/Tween (Promega). Goat serum blocking solution (Vector Laboratories, diluted 1:10 with PBS) was added, and samples were incubated for 1 hour at room temperature, followed by 2 rinses with PBS/Tween. Ly6G rat anti-mouse antibody (BD Biosciences, diluted 1:200 with PBS) was added, with 1 sample remaining as a control using unlabelled immunoglobulin G antibody (BD Biosciences). Samples were incubated for 1 hour at room temperature before the second antibody (Alexa Fluor 546 goat antirat, Life Technologies, diluted 1:1000 with PBS) was applied to all samples. After 1 hour of incubation in the dark, 4',6-diamidino-2-phenylindole (DAPI) (Carl Roth) was added, and the samples were coverslipped.

Microscopy was performed using an Axio Vert.A1 microscope (Carl Zeiss). Neutrophils were detected by Ly6G positivity, and nuclei by DAPI positivity. Images were taken at 20× and 40× magnification. In addition, panoramic images were taken by combining 20× images to show the entire thrombus.

Panoramic images were analyzed using Fiji (National Institutes of Health) by subtracting background noise and measuring the mean intensity of the Ly6G and DAPI signals.

### 2.9 Statistical analysis

Statistical analysis was performed using GraphPad Prism 8 for macOS (GraphPad Software). Dichotomous variables were expressed as sum (%), and continuous variables as mean ± SD, represented in the graphs as mean ± SEM to illustrate the precision of the estimated mean and to facilitate comparability between groups. Dichotomous variables were compared using the chi-square test or Fisher’s exact test when expected cell size was <5. The normal distribution of continuous variables was evaluated graphically using Q-Q plots. Normally distributed data were compared using one-way analysis of variance, and nonnormally distributed data were compared using the Kruskal–Wallis test. A P value < .05 (∗) was considered statistically significant.

## 3 Results

**Figure 1:**
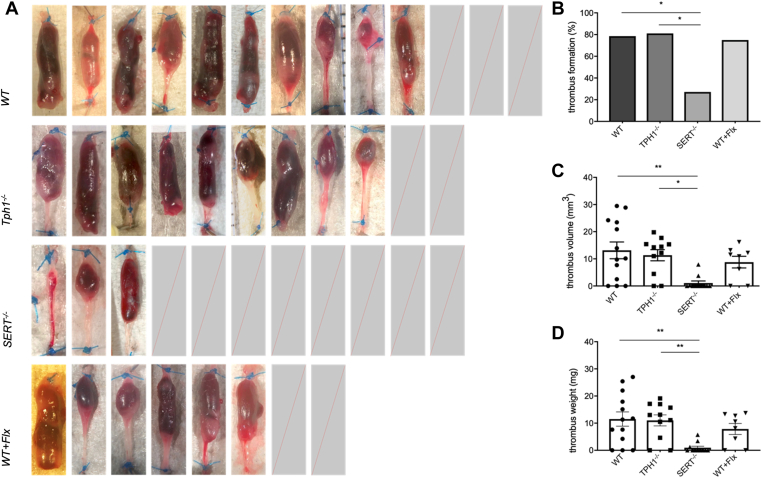
Serotonin transporter knockout (SERT^-/-^) mice formed significantly fewer and smaller thrombi compared with wild-type (WT) and tryptophan hydroxylase 1 knockout (Tph1^-/-^) mice. Thrombus formation after 48 hours in the vena cava below the stenosis in WT, Tph1^-/-^, SERT^-/-^, and WT + fluoxetine (Flx) mice. Variety of thrombi in appearance, volume, and shape, as well as the mice undergoing surgery without thrombus formation (A). The frequency of thrombus formation was expressed as a percentage of all operated animals and compared between groups (B). Thrombus volume (C) and weight (D) were measured and then compared among the 4 groups of mice. Data are mean ± SEM, ∗*P* < .05, ∗∗*P* < .005, *n* = 8 to 13.

Thrombus formation was detected in 78.6% (10/13) of the WT group, 81.1% of the Tph1^-/-^ group (9/11), and 75% (6/8) of the WT + Flx group (Figure 1A). In the SERT^-/-^ group, thrombus formation was seen in 27.3% of mice (3/11). Thrombi formed significantly less frequently in the SERT^-/-^ group compared with the WT group (27.3% vs 78.6%; *P* = .038; Figure 1B) and the Tph1^-/-^ group (27.3% vs 81.1%; *P* = .03; Figure 1B). SERT^-/-^ mice tended to have less thrombus formation compared with WT + Flx mice (27.3% vs 75%; *P* = .07; Figure 1B).

Volumes of thrombi varied from 0 to 27.7 mm^3^ in WT mice, 0 to 19.1 mm^3^ in Tph1^-/-^ mice, and 0 to 16.2 mm^3^ in WT + Flx mice. SERT^-/-^ mice showed a smaller range of volumes, from 0 to 7.9 mm^3^.

There was a significant difference in mean volume of thrombi in WT mice compared with SERT^-/-^ mice (mean ± SD, 13.1 mm^3^ ± 11.1 vs 1.09 mm^3^ ± 2.53; P = .002; Figure 1C). Thrombi of Tph1^-/-^ mice were significantly larger compared with SERT^-/-^ mice (11.3 mm^3^ ± 6.84 vs 1.09 mm^3^ ± 2.53; P = .02; Figure 1C). No significant volume difference was seen between the WT group and the Tph1^-/-^ group, as well as the WT + Flx group.

A significant difference in thrombus weight was detected between WT and SERT^-/-^ mice (mean ± SD, 11.5 mg ± 9.48 vs 0.94 mg ± 1.89; *P* = .003; Figure 1D) and between Tph1^-/-^ and SERT^-/-^ mice (11.0 mg ± 6.73 vs 0.94 mg ± 1.89; *P* = .006; Figure 1D), whereas the comparison of thrombus weight between WT and Tph1^-/-^ mice and WT and WT + Flx mice was not significantly different.

**Figure 2:**
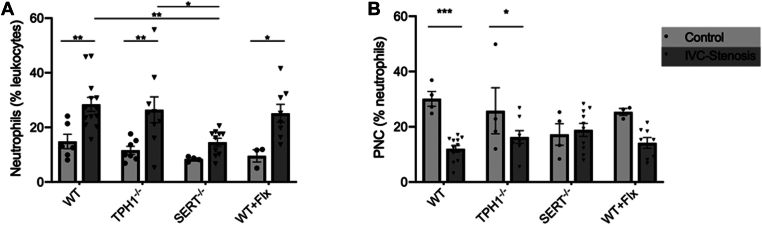
In serotonin transporter knockout (SERT^-/-^) mice, there is no significant increase in neutrophils in peripheral blood 48 hours after surgery compared with control animals without any surgery. Percentage of neutrophils in peripheral blood between mice that underwent inferior vena cava (IVC) stenosis and control mice, and among the different groups of mice after IVC stenosis (A). Proportion of neutrophils forming complexes with platelets between mice that underwent vena cava stenosis and control mice, and among the different groups of mice after IVC stenosis (B). Data are mean ± SEM, ∗*P* < .05, ∗∗*P* < .005, ∗∗∗*P* < .0005, *n* = 8 to 13. PNC, platelet-neutrophil complex; TPH1^-/-^, tryptophan hydroxylase 1 knockout; WT, wild-type; WT + Flx, wild-type + fluoxetine.

There was a significant difference in the proportion of neutrophil granulocytes in the leukocyte population (in %) in mice that underwent vena cava stenosis compared with control mice in the WT, Tph1^-/-^, and WT + Flx groups (Figure 2A) in peripheral blood. In the WT group, the operated mice showed a significantly higher proportion of neutrophil granulocytes to leukocytes compared with the control mice (mean ± SD, 28.4% ± 9.19% vs 14.9% ± 6.44%; *P* = .008; Figure 2A). The percentage of neutrophil granulocytes was significantly higher in Tph1^-/-^ mice undergoing IVC stenosis (26.4% ± 14.2% vs 11.6% ± 3.91%; *P* = .004; Figure 2A), as well as in WT + Flx mice (25.2% ± 9.28% vs 9.56% ± 3.88%; *P* = .036; Figure 2A), compared with control groups. SERT^-/-^ mice showed no significant difference in the percentage of neutrophil granulocytes to total leukocytes between mice undergoing IVC stenosis and control animals (14.6% ± 4.4% vs 8.4% ± 0.84%; *P* = .64; Figure 2A).

The comparison between mice groups undergoing IVC stenosis revealed a significantly higher percentage of neutrophil granulocytes (% of leukocytes) in WT mice compared with SERT^-/-^ mice (mean ± SD, 28.4% ± 9.19% vs 14.6% ± 4.4%; *P* = .007; Figure 2B), as well as in Tph1^-/-^ mice compared with SERT^-/-^ mice (26.4% ± 14.2% vs 14.6% ± 4.4%; *P* = .035; Figure 2B). There was no significant difference in the other groups (Figure 2B).

In WT and Tph^-/-^ mice, the percentage of platelet-neutrophil complexes (PNCs) in the circulation decreased significantly after IVC stenosis compared with their respective controls (mean ± SD, 12.05% ± 4.27% vs 30.1% ± 5.31%; *P* = .0003; Figure 2C). After induction of IVC stenosis, circulating PNCs did not differ significantly between mice in all treatment groups (Figure 2D).

Bleeding time after tail tip amputation was assessed in 8 WT (range, 69-455 seconds), 8 Tph1^-/-^ (range, 220-900 seconds), 8 SERT^-/-^ (range, 94-685 seconds), and 9 WT + Flx (range, 89-900 seconds) mice.

**Figure 3:**
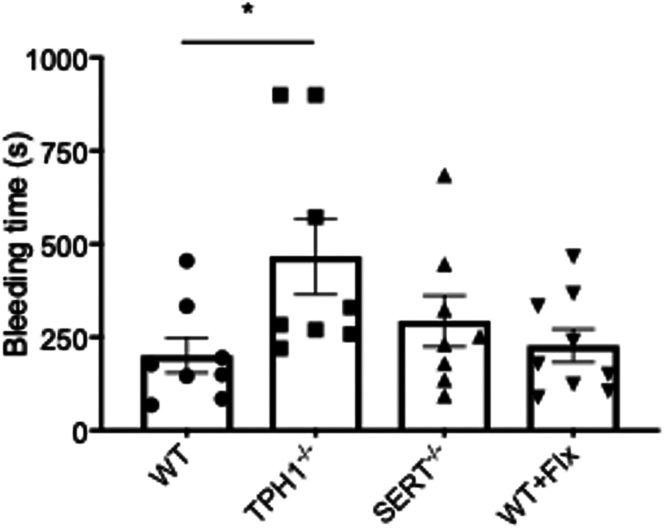
The complete absence of peripheral serotonin (tryptophan hydroxylase 1 knockout [Tph1^-/-^]) is associated with extended bleeding time in mice. Bleeding time after tail tip amputation in seconds (s) between wild-type (WT), Tph1^-/-^, serotonin transporter knockout (SERT^-/-^), and WT + fluoxetine (Flx) mice. Data are mean ± SEM, ∗*P* < .05, *n* = 8 to 9.

Mean bleeding time was significantly longer in Tph1^-/-^ mice compared with WT mice (mean ± SD, 467 ± 288.2 vs 201.5 ± 130.5 seconds; *P* = .03; Figure 3). Bleeding time in WT mice was comparable to SERT^-/-^ mice (201.5 ± 130.5 vs 293.6 ± 192.9 seconds; *P* = .58) and WT + Flx mice (201.5 ± 130.5 vs 295.4 ± 246.6 seconds; *P* = .78).

**Figure 4:**
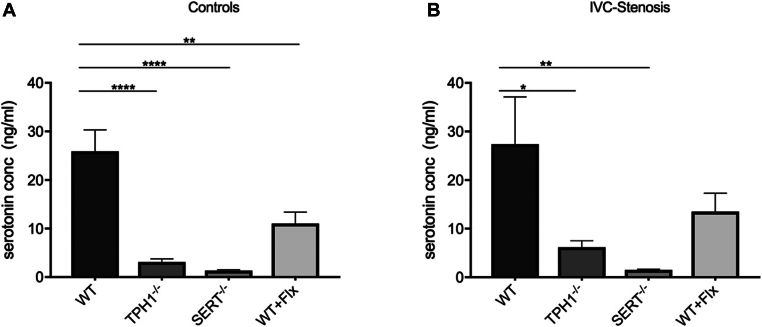
Plasma serotonin concentration (conc) in control (A) and operated animals (B). Data are mean ± SEM. ∗*P* < .05, ∗∗*P* < .01*, ∗∗∗∗P* < .0001, *n* = 8 to 16. TPH1^-/-^, tryptophan hydroxylase 1 knockout; SERT^-/-^, serotonin transporter knockout; WT, wild-type; WT + Flx, wild-type + fluoxetine.

Plasma serotonin concentration was comparable in mice that underwent surgery and control animals. WT mice revealed the highest concentration in both the control group (mean ± SD, 25.9 ng/mL ± 17.6; Figure 4A) and the group that underwent surgery (24.7 ng/mL ± 30.1; Figure 4B). Plasma serotonin concentration in Tph1^-/-^ (3.13 ng/mL ± 2.36; *P* < .0001), SERT^-/-^ (1.35 ng/mL ± 0.51; *P* < .0001), and WT + Flx control mice (11.1 ng/mL ± 8.02; *P* = .003) was significantly lower compared with WT controls.

**Figure 5:**
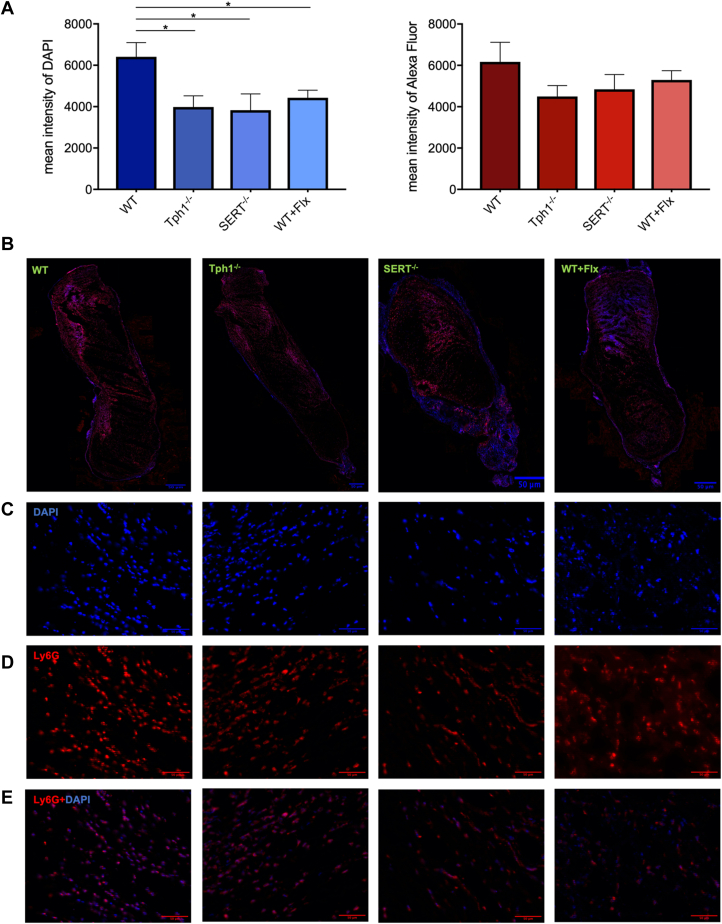
Immunofluorescence staining of mouse thrombi within the inferior vena cava of wild-type (WT), tryptophan hydroxylase 1 knockout (Tph1^-/-^), serotonin transporter knockout (SERT^-/-^), and WT + fluoxetine (Flx) mice. Representation of the mean intensity of lymphocyte antigen 6 complex locus G6D (Ly6G; red) and 4',6-diamidino-2-phenylindole (DAPI; blue) of the whole thrombus (A). Creation of panoramic images of the entire thrombus from multiple small images at 20× magnification (B). Visualization of representative areas of the thrombus at 20× magnification in DAPI staining (C), the same section in Ly6G staining (D), and superimposition of both channels (E). Scale for all images is 50 μm. Data are mean ± SEM, ∗P < .05, n = 3 to 7.

DAPI intensity was significantly higher in WT mice compared with Tph1^-/-^ mice (mean ± SD, 6407 ± 1684 vs 3980 ± 1326; *P* = .018), SERT^-/-^ mice (6407 ± 1684 vs 3828 ± 1582; *P* = .02), and WT + Flx mice (6407 ± 1684 vs 4433 ± 961; *P* = .048; Figure 5A).

The mean intensity of Ly6G on neutrophils was not significantly different between WT, Tph1^-/-^, SERT^-/-^, or WT + Flx mice. Overall, the highest intensity of Alexa Fluor 546, indicating Ly6G, was observed in WT mice and the lowest in Tph1^-/-^ mice (Figure 5A).

## 4 Discussion

This study, using a vena cava stenosis model, aimed to examine venous thrombus formation in mice with focus on the influence of peripheral platelet serotonin. To minimize experimental setup changes, the surgical procedures were conducted by the same surgeon using standardized methods and materials. Mice were male and similar in age and weight. During the surgery, all visible side and back branches of the vena cava were ligated to ensure consistent initial conditions. This was done to eliminate the confounding factor of open branches, which could reduce thrombus volume [16,18]. However, it should be noted that the mouse model used in this study lacks venous valves and a muscle pump, which are present in the human venous system [16]. We found that up to 35% of the cases showed a lack of thrombus formation, which is consistent with previous studies using the same method [16,19]. Moreover, thrombus volume varied within each group. Notably, the group of mice lacking SERT exhibited a significantly higher rate of absent thrombus formation.

The SERT^-/-^ group developed the smallest and lightest thrombi compared with the WT and Tph1^-/-^ groups. Thus, the presence or absence of peripheral serotonin, available in circulating platelets, seems to have no or little effect on the development of venous thrombosis. However, a deficiency in SERT on platelets (and other cells like dendritic cells, monocytes, macrophages, mast cells, lymphocytes, and endothelial cells [[20], [21], [22], [23]]) results in a reduction in thrombus formation within the venous system.

Neutrophils represent a crucial cell type in this context. They play a pivotal role in the process of immunothrombosis, where they initially adhere to the endothelium and subsequently recruit other immune cells and platelets [2,4,24]. There is several evidence that serotonin promotes leukocyte recruitment in inflammation [9,13,20,25,26]. Additionally, they facilitate the formation of blood clots by releasing NETs [4,27]. In the WT, Tph1^-/-^, and WT + Flx groups, neutrophil counts in the blood increased 48 hours after surgery compared with control animals. There was no increase in neutrophils and no significant thrombus formation in the SERT^-/-^ group. The recruitment of neutrophils, for instance, from the spleen and bone marrow can be attributed to the subsequent inflammation of venous thrombosis. It is already known that the highest increase in neutrophils occurs after approximately 48 hours [28]. In this study, the blood was analyzed after this time period. In the absence of SERT, less neutrophil recruitment and reduced formation of venous thrombi were observed.

This leads to the assumption that different serotonin concentrations in platelets, particularly the absence of SERT, result in reduced inflammation and, thus, a lack of or reduced thrombus formation. This effect may be due to an as-yet unknown distribution of SERT on neutrophils.

It is also possible that cells expressing SERT (such as monocytes, macrophages, and endothelial cells) have a thrombogenic effect depending on serotonin, which has not yet been confirmed. Furthermore, many of the cells expressing SERT also express Tph1 (such as monocytes, macrophages, mast cells, and endothelial cells) and are therefore able to synthesize serotonin themselves [14,15,29]. These cells do not experience serotonin depletion due to the absence of SERT. These and other SERT-expressing cells, involved in venous thrombus formation, require further investigation.

We assumed that results similar to those observed in the SERT^-/-^ mice would also be expected in the pharmacologic counterpart, the Flx fed animals, where SERT is selectively blocked >21 days to deplete platelet serotonin pools, as the lifespan of a platelet is about 7 days, as described before [13]. However, our data indicate no significant difference between the WT + Flx mice and WT and Tph1^-/-^ mice, although there is a trend toward smaller thrombi and less neutrophil recruitment. Blocked SERT by Flx results in a reduced uptake and storage capacity of serotonin in platelets, which leads to reduced overall serotonin production [30]. This phenomenon can also be assumed in SERT^-/-^ mice. In addition, SERT^-/-^ mice are SERT-deficient since birth, thus preventing platelets and megakaryocytes from ever taking up serotonin during their formation and maturation. However, in mice fed Flx for only 3 weeks, serotonin uptake into platelets and megakaryocytes was possible until Flx accumulated in plasma, resulting in the residual presence of serotonin in maturing platelets and the detection of additional serotonin in plasma [31]. Serotonin is not only stored as a hormone in granules to be released, but it can also be bound to proteins, a process called serotonylation [7]. This process was possible before treatment with Flx, but most likely not in SERT deficiency from birth.

The concentration of circulating PNCs was lower in the WT, Tph1^-/-^, and WT + Flx groups undergoing surgery than in the respective controls. In contrast, in SERT^-/-^ mice, the percentage of circulating PNCs did not increase after surgery. It can be assumed that the PNCs in the thrombosis-forming groups are so closely involved in the formation of thrombosis, or even initiate and reinforce it [4,32] that they are less detectable in the circulating blood. The observation that PNCs are similar in operated SERT^-/-^ mice and their controls may indicate that, due to reduced inflammation and neutrophil recruitment, fewer "activated" neutrophils and platelets are generally involved in these complexes. Furthermore, there are small or no thrombi in which the complexes can be contained. Thus, they are still detectable in the peripheral blood as free circulating complexes.

We suggest that the reduction in circulating platelet-leukocyte complexes postsurgery may be due to their incorporation into the thrombus. However, this theory could be substantiated with data from thrombus material and the complexes contained therein. Nevertheless, the complexes could not be reliably discriminated in the thrombus, as they do not necessarily remain in their original conformation during thrombus formation and their further activation. Activation of neutrophils within the thrombus is often followed by NETosis, which propagates coagulation and thrombus growth [4,32,33].

Furthermore, the bleeding time was examined to ascertain whether primary hemostasis might influence the difference in thrombus formation. The complete absence of serotonin in platelets, resulting from the knockout of Tph1 and the subsequent lack of serotonin synthesis in the periphery, leads to a prolonged bleeding time due to reduced platelet aggregation, as previously described [7].

In our study, mice in the SERT^-/-^ and WT + Flx groups demonstrated surprisingly no extended bleeding time compared with the WT mice. It was previously described that these mice have a mild bleeding phenotype due to a decrease in serotonin receptor expression [34]. It must be noted that our WT group showed longer bleeding times of 201.5 ± 130.5 seconds compared with the control mice in a previous study by Oliver et al. [35] (about 100 seconds), but bleeding times of SERT^-/-^- and Flx-treated mice seem comparable in our study and the study by Oliver et al. [35].

On the one hand, this study, as well as previously published studies from other groups, show an increased bleeding tendency in the absence of platelet serotonin [7]. On the other hand, the Tph1^-/-^ group had the largest and most frequent venous thrombosis in this study. This indicates that venous thrombogenesis, in contrast to arterial thrombogenesis, is more dependent on the interaction of immune cells than on hemostasis and the underlying activation and aggregation of platelets. This confirms the hypothesis of a much more complex interplay among different cells, alongside platelets, and highlights the importance of immunothrombosis in the venous system.

Immunofluorescence staining of neutrophils in thrombus tissue was used to assess and quantify neutrophil migration and distribution into the thrombus. The mean intensity of nucleated cells and migrated neutrophils was highest in the thrombi of the WT group. The fact that platelet serotonin promotes the migration of neutrophils into inflamed tissue is already known [13] and supports the increased occurrence of cells in the WT thrombi. Nevertheless, the thrombi in the Tph1^-/-^ mice were comparable in size. Thus, there seems to be another previously unknown mechanism in the development of venous thrombosis that is independent of serotonin. Near-absence of thrombus formation in the SERT^-/-^ group points toward a mechanism related to the expression of SERT. This may also be possible in other immune cells, such as monocytes, macrophages, mast cells, or endothelial cells themselves.

Histologic analysis suggests that increased neutrophil migration into the thrombus has a positive effect on thrombus size, but that this migration is not solely caused by differences in serotonin levels in platelets. SERT deficiency also appears to affect neutrophil migration and activation, and consequently, thrombus size.

## 5 Conclusion

The surprising finding that thrombus formation is impaired in the absence of SERT but not in the absence of total peripheral serotonin released from platelets or pharmacologic SERT blocking needs to be confirmed by further research on the distribution of SERT on neutrophils. Furthermore, the involvement of other SERT-expressing cells, such as monocytes, macrophages, mast cells, or endothelial cells, needs to be examined with focus on their serotonergic effects.
