# Supplementary material for: Serotonin transporter deficiency, but not absence of platelet serotonin, impairs thrombus formation in a model of deep vein thrombosis
Source: Res Pract Thromb Haemost. 2025 Jul 15;9(5):102970. doi: 10.1016/j.rpth.2025.102970 (PMC12351330; doi:10.1016/j.rpth.2025.102970)
Supplement: Supplementary Material [file mmc1.docx]

Supplementary Material


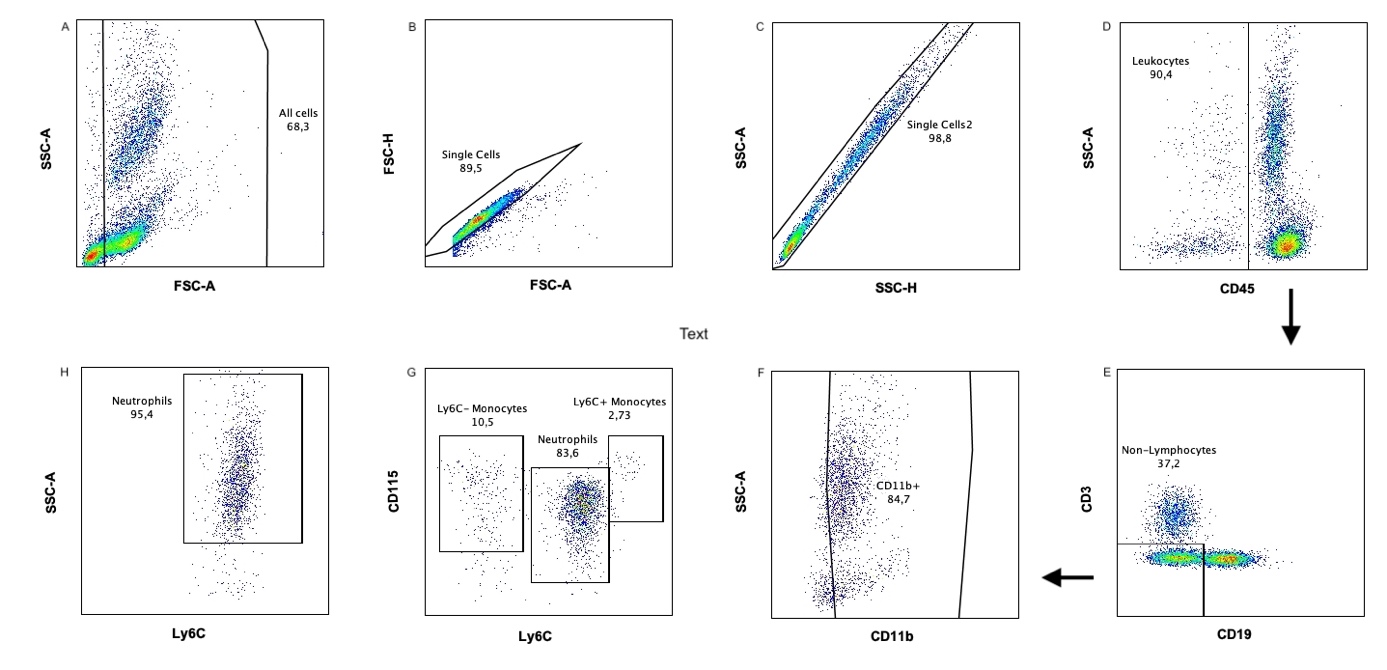


Suppl. Figure: Flow cytometric dot plots of mouse blood 48h after induction of deep vein thrombosis. Identification of all leukocytes (D), monocytes (G) and free neutrophils (H).


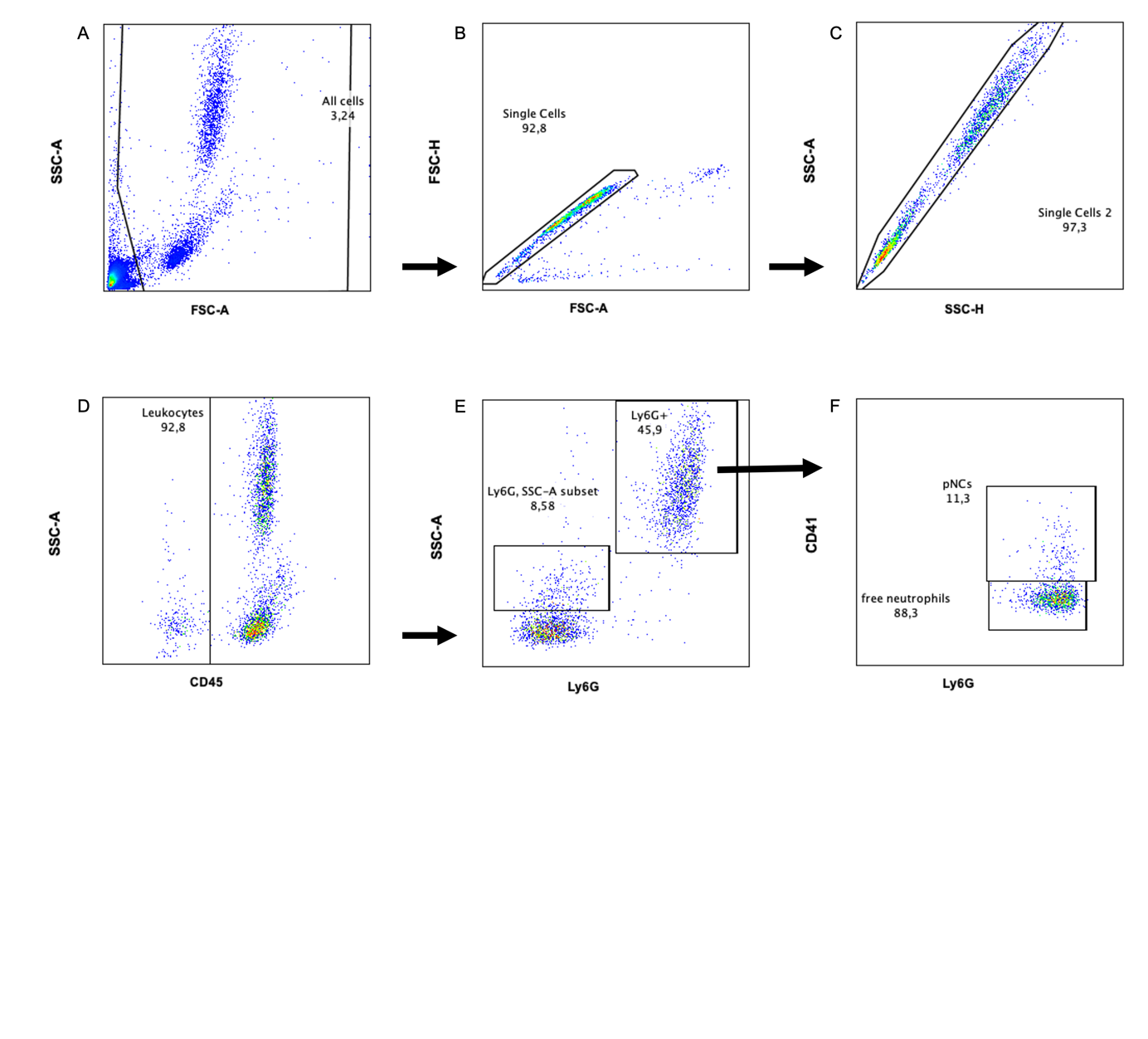


Suppl. Figure: Flow cytometric dot plots of mouse blood 48h after induction of deep vein thrombosis. Identification of all leukocytes (D), all neutrophils (E) and platelet-neutrophil-complexes (F).
